# Does Prenatal Exposure to CNS Stimulants Increase the Risk of Cardiovascular Disease in Adult Offspring?

**DOI:** 10.3389/fcvm.2021.652634

**Published:** 2021-03-04

**Authors:** Boyd R. Rorabaugh

**Affiliations:** Department of Pharmaceutical Science, Marshall University School of Pharmacy, Huntington, WV, United States

**Keywords:** prenatal stimulants, methamphetamine, cocaine, nicotine, epigenetic, cardiovascular disease in adult offspring

## Abstract

Prenatal exposure to an adverse uterine environment can have long lasting effects on adult offspring through DNA methylation, histone acetylation, and other epigenetic effects that alter gene expression and physiology. It is well-known that consumption of CNS stimulants such as caffeine, nicotine, amphetamines, and cocaine during pregnancy can adversely impact the offspring. However, most work in this area has focused on neurological and behavioral outcomes and has been limited to assessments in young offspring. The impact of prenatal exposure to these agents on the adult cardiovascular system has received relatively little attention. Evidence from both animal and human studies indicate that exposure to CNS stimulants during the gestational period can negatively impact the adult heart and vasculature, potentially leading to cardiovascular diseases later in life. This review discusses our current understanding of the impact of prenatal exposure to cocaine, methamphetamine, nicotine, and caffeine on the adult cardiovascular system.

## Introduction

Drug and alcohol use during pregnancy is a significant public health concern. The 2018 National Survey on Drug Use and Health estimated that 5 % of pregnant women used an illicit drug within the past month (1). Use of tobacco products (12%) and binge consumption of alcohol (5%) are also common among pregnant women (1). Prenatal exposure to these substances can have a profound impact on adult offspring. It is well-established that substance use during pregnancy can negatively impact cognition (2, 3), the stress response (4), anxiety (5, 6), and susceptibility to drug addiction (7, 8) in the adult offspring. These transgenerational effects involve epigenetic mechanisms (DNA methylation, histone acetylation, micro RNA) (9–11) as well as drug-induced changes in endocrine function (12, 13), receptor expression (14–16), and structural changes within the brain (17, 18) that can have pathological consequences extending into adulthood.

The premise of the “developmental origins of adult health and disease” (DOAHD) hypothesis, originally proposed by de Boo and Harding (19), is that the developing fetus responds to an adverse uterine environment (in the form of malnutrition, fetal hypoxia, exposure to environmental toxins, etc.) through physiological adaptations that include epigenetic changes in gene expression (“fetal reprogramming”). These changes in gene expression and physiology persist into adulthood and can increase the chances of developing cardiovascular disease, diabetes, and other disorders later in life. There is growing evidence that prenatal exposure to central nervous system (CNS stimulants) can lead to adverse consequences in the adult. Most studies exploring the impact of prenatal exposure to CNS stimulants have focused on behavioral and neurological outcomes. However, recent studies indicate that prenatal exposure to CNS stimulants such as methamphetamine, cocaine, nicotine, and caffeine, can lead to diabetes (13, 20), obesity (20), alterations in lipid metabolism (21–23), vascular dysfunction (24–26), and increased susceptibility to cardiac ischemic injury (27, 28) in adult offspring. The purpose of this review is to summarize our current understanding of the impact of prenatal exposure to CNS stimulants on adult cardiovascular function. Our understanding of the prenatal impact of CNS stimulants on the heart and vasculature of adult offspring is heavily dependent on animal models. However, data from human studies are included in this review to the extent that they are available.

## Impact of Prenatal Exposure to CNS Stimulants on the Adult Cardiovascular System

### Cocaine

Cocaine increases adrenergic, dopaminergic, and serotonergic signaling in the CNS and peripheral organs by blocking the removal of norepinephrine, dopamine, and serotonin from the synapse through their respective transporters. Maternal cocaine use acutely increases blood pressure, heart rate, and catecholamine levels in both the mother and the fetus (29), and there is evidence that prenatal exposure to cocaine produces long-lasting effects in the cardiovascular system that extend into adulthood. Zhang et al. reported hypertrophy and increased apoptosis in neonatal cardiomyocytes from rats that had been prenatally exposed to cocaine (30). When the animals became adults the males developed myocardial hypersensitivity to ischemic injury (28). Importantly, prenatal cocaine had no impact on sensitivity to ischemia in their female littermates, indicating that this effect was sex dependent. Subsequent work demonstrated that the cardioprotective benefit of ischemic preconditioning (the process by which exposure to brief periods of sublethal ischemia protects the heart from injury induced by a subsequent episode of prolonged ischemia) was abolished in adult male rats (but not their female littermates) that had prenatally exposed to cocaine (31). Myocardial hypersensitivity to ischemia in these animals was the result of cocaine-induced methylation of the promoter of the gene encoding protein kinase C-ε (PKC-ε) and a subsequent decrease in the expression of this cardioprotective protein (32, 33). These data demonstrate that cocaine can induce sex-dependent epigenetic changes during the gestational period and can increase the heart's vulnerability to ischemic injury during adulthood.

Prenatal exposure to cocaine also alters adult vascular function. Xiao et al. (34) found that prenatal cocaine exposure had no effect on basal blood pressure in adult rats, but norepinephrine-induced increases in blood pressure were significantly potentiated in these animals. Consistent with this response, norepinephrine-induced vasoconstriction was also potentiated in mesenteric resistance arteries, and myofilament sensitivity to calcium was increased in these vessels (34). Furthermore, prenatal exposure to cocaine resulted in sex dependent (male only) attenuation of endothelium-dependent relaxation. The baroreflex was also suppressed in adult male rats following prenatal exposure to cocaine. These data demonstrate that fetal exposure to cocaine induces changes in both the endothelium and vascular smooth muscle that alter vascular function and may increase the risk of developing hypertension.

Prenatal cocaine also disrupts the function of the adult coronary vasculature. Coronary microvessels constrict and dilate in response to changes in luminal pressure. These pressure-induced changes in myogenic tone provide an important mechanism for the autoregulation of blood flow within the myocardium (35, 36). However, the ability of coronary arteries to regulate myogenic tone in response to changes in arterial pressure is disrupted in adult rats following fetal exposure to cocaine (26). The ability of prenatal cocaine to alter vascular function is not unique to rodents. Potentiation of norepinephrine-, endothelin-, and serotonin-induced vasoconstriction have been reported in the cerebral vasculature of piglets that were prenatally exposed to cocaine (37, 38). These studies provide evidence that prenatal exposure to cocaine can have long term effects on the vasculature that may increase the risk of developing hypertension or other vascular disorders later in life.

Studies of blood pressure in humans that were prenatally exposed to cocaine are sparse and have produced mixed results. Some investigators have reported elevated blood pressure in children following prenatal exposure to cocaine (39, 40), while others have found no effect (41, 42). We are unaware of any studies that have assessed blood pressure, the prevalence of ischemic heart disease, or other vascular outcomes in adult humans with documented prenatal exposure to cocaine. In light of the fact that cardiovascular disease is much more prevalent in adults than in children, the lack of human studies involving adults that were prenatally exposed to cocaine represents a significant gap in our understanding of the impact of prenatal exposure to cocaine on adult cardiovascular function.

### Methamphetamine

The Infant Development, Environment, and Lifestyle (IDEAL) study estimated that 5% of pregnant women used methamphetamine at some point during their pregnancy (43). Unfortunately, this study also found that only 36% of women who used methamphetamine during pregnancy decreased the frequency of the drug's use between the first and third trimesters. Methamphetamine use among the remaining women was either unchanged (55%) or increased (9%) over the course of pregnancy, resulting in continued methamphetamine exposure to their unborn children (44).

Methamphetamine and cocaine differ in their mechanisms of action, but both agents produce similar physiological effects that are mediated by increased adrenergic and dopaminergic signaling. Thus, it is not surprising that prenatal exposure to methamphetamine produce outcomes in the hearts of adult offspring that are similar to those observed following prenatal exposure to cocaine. Our laboratory found that adult rats that were prenatally exposed to methamphetamine develop myocardial hypersensitivity to ischemia (27). Similar to Zhang's work with prenatal cocaine (28, 45), we found that PKC-ε expression was significantly decreased in adult rats that had been prenatally exposed to methamphetamine, suggesting that prenatal exposure to these drugs might alter myocardial sensitivity to ischemia in the adult heart through a similar (PKC-ε-dependent) mechanism. However, the cardiac effect of prenatal methamphetamine differed from that of cocaine with respect to sex-dependence. Prenatal methamphetamine selectively suppressed PKC-ε expression and sensitized the heart to ischemia exclusively in female offspring (27) while cocaine induced these effects only in males (28, 45). Although this review focuses on the effects of stimulant exposure during the prenatal period, it should be noted that female rats (but not male rats) treated with methamphetamine during early adulthood also develop sex-dependent myocardial hypersensitivity to ischemia (46). Furthermore, this effect persists following at least 1 month of subsequent abstinence from the drug (46), indicating that methamphetamine exposure either during gestation or during adulthood produces changes in the adult heart that are long-lasting and potentially irreversible. In addition to direct effects on the heart, methamphetamine exposure during gestation may promote the development of cardiovascular risk factors such as diabetes, hypertension, and obesity. A recent study by Korchynska et al. (13) reported hypermethylation and decreased expression of multiple genes required for insulin production by pancreatic β cells in adult mice that were prenatally exposed to methamphetamine, amphetamine, or cocaine. This resulted in permanent impairment of insulin production and lifelong dysregulation of glucose homeostasis. In addition, increased body weight and elevated blood pressure have been reported in children born to mothers who used methamphetamine during pregnancy (40). Studies in this area have been limited to laboratory animals and children. It is unknown whether men and women who were exposed to these stimulants during gestation are at increased risk of developing diabetes, obesity, hypertension, or other cardio-metabolic disorders.

It is unclear why fetal exposure to methamphetamine produces sex dependent effects in the hearts of adult offspring. Shen et al. (47) reported disruption of the hypothalamic-pituitary-ovarian axis in women who chronically used methamphetamine. This suggests that prenatal exposure to methamphetamine may disrupt ovarian function, resulting in a loss of estrogen-dependent cardioprotection. Alternatively, the sex-dependent effect may result from differences in tissue distribution. Methamphetamine concentrations in the brain and serum have been reported to be significantly higher in adult female rats compared to adult males following a subcutaneous injection of methamphetamine (48). It is possible that similar sex differences in the tissue distribution of methamphetamine also exist in the male and female fetus following transplacental delivery, resulting in sex-dependent outcomes during adulthood. Alternatively, the sex-dependent effects of prenatal exposure to methamphetamine or cocaine might result from sex differences in the dynamics of neurotransmitter release and reuptake. Adult female rats have greater dopamine transporter (DAT) expression than males in some regions of the brain (striatum and substantia nigra) (49, 50) and exhibit higher rates of striatal dopamine release and dopamine reuptake than males (51). Thus, sex differences in neurotransmitter release and reuptake or sex differences in the expression of adrenergic and dopaminergic receptors in the developing fetus could underlie the sex-dependent effects of prenatal exposure to these CNS stimulants. This hypothesis is speculative as we are unaware of any studies that have compared the expression levels of DAT, the norepinephrine transporter (NET), or adrenergic and dopaminergic receptors in the developing male and female fetus. Understanding the mechanism by which prenatal exposure to methamphetamine or cocaine induces sex-dependent effects in the adult heart is complicated by the fact that some methamphetamine induced effects are found exclusively in males (52) and other effects occur exclusively in females (13, 27). Similarly, prenatal cocaine produces some effects in only in male offspring (26, 28) and other effects that occur exclusively in female offspring (13, 26). Further work is needed to understand the mechanism by which these effects occur in a sex-dependent manner.

### Nicotine

Cigarette smoking has dramatically decreased in the United States since reaching its peak in the 1950s (53). However, the 2018 Survey on Drug Use and Health found that 21.5% of US residents over the age of 12 had used tobacco products (including cigarettes, cigars, pipe tobacco, and smokeless tobacco) within the past month (54). Nicotine readily crosses the placenta and achieves a concentration in fetal serum that is similar to that of maternal circulation (55). The increased risk of vascular disorders among smokers is well-established. However, the impact of prenatal exposure to tobacco on the adult vasculature has received relatively little attention. Significant increases in basal blood pressure have been reported in adult rats (56, 57) and mice (58) following prenatal exposure to nicotine. Prenatal exposure to nicotine potentiates norepinephrine- and angiotensin II-induced increases in blood pressure (59, 60) and enhances vasoconstriction of mesenteric resistance arteries (25, 60) in adult rats. These nicotine-induced effects are associated with changes in the expression of angiotensin II receptors in vascular smooth muscle (60), increased thickening of the media of the arterial wall (60), enhanced sensitivity of vascular smooth muscle to calcium (60), increased oxidative stress (24, 59), attenuation of endothelium-dependent relaxation (24), and disruption of perivascular adipose-dependent mechanisms that regulate vascular tone (56, 61). Attenuation of the baroceptor reflex and increased basal levels of circulating catecholamines and angiotensin II have also been reported in adult mice and rats following prenatal exposure to nicotine (58, 60, 62, 63). Thus, prenatal nicotine may increase blood pressure through mechanisms that involve targets within the endothelium, vascular smooth muscle, and perivascular adventitia as well as mechanisms involving neuronal and hormonal regulation.

Human studies assessing blood pressure in children born to mothers who used tobacco while pregnant have produced mixed results. Some investigators have found elevated blood pressure in these children (64–67) while others have reported no significant effect (68, 69). Studies in adult offspring are limited. A study in Sweden (using data from the Swedish birth registry and blood pressure measurements taken from 85,000 young men who registered for compulsory military service) found elevated blood pressure in young men who were born to mothers that smoked during pregnancy (70). Although statistically significant, this increase in blood pressure did not reach the level required for a diagnosis of hypertension. Other investigators have found that adult women born to mothers who smoked during pregnancy were more likely to develop obesity and hypertension than women born to non-smoking mothers (71). These data suggest that people who were exposed to tobacco during the gestational period might be at increased risk of developing cardiovascular risk factors later in life.

Animal studies indicate that prenatal nicotine exposure also produces long term effects on the heart. Lawrence et al. (72) found that adult male and female rats that were prenatally exposed to nicotine had larger infarcts and attenuated postischemic recovery of contractile function following exposure to an ischemic insult. Similar to cocaine (31, 33) and methamphetamine (27), rats that were prenatally exposed to nicotine also exhibit decreased expression of cardioprotective PKC-ε (72–74). Other investigators have reported increased heart rates, decreased contractile function (stroke volume, fractional shortening, and ejection fraction), fibrosis, and thickening of the left ventricular walls in adult rats following prenatal exposure to nicotine (62, 63). These preclinical studies provide evidence that maternal use of tobacco increases the risk of ischemic injury, suppresses cardiac contractile function, and induces cardiac remodeling in adult offspring.

### Caffeine

Caffeine is the most commonly consumed CNS stimulant in the United States. Caffeine is consumed by 89 % of the US population (typically in the form of soft drinks, energy drinks, coffee, tea, or chocolate) on any given day (75). Importantly, pregnancy significantly prolongs the half-life of caffeine from 3–4 h in non-pregnant women to 8–10 h during the last month of pregnancy (76–78). Cyp1A2, the primary enzyme that metabolizes caffeine, is not expressed in the human placenta, fetus, or the neonate (79, 80) resulting in a fetal half-life of 52–95 h (81, 82). Because of the slow rate of fetal elimination of caffeine, regular daily consumption can lead to its accumulation in the fetus. Caffeine blocks A1 and A2 adenosine receptors at concentrations that are typically achieved through dietary consumption, although high concentrations of caffeine can also inhibit phosphodiesterase activity (83).

Animal studies indicate that caffeine exposure during the prenatal period induces epigenetic changes in gene expression that result in cardiac hypertrophy (characterized by increased ventricular mass, increased ventricular wall thickness, and decreased stroke volume) in adult offspring (84–86). Wendler's group found that mice born to pregnant females that had been injected with caffeine during gestational days 6.5–9.5 had a significant overall decrease in DNA methylation, and they identified over 7,700 sites of differential methylation in the genome (85). Consistent with the cardiac phenotype of these animals, many of the areas of differential methylation were located in genes associated with cardiac hypertrophy and cardiomyopathy. These changes did not occur in adult mice exposed to caffeine during gestational days 10.5–13.5, indicating that these caffeine-induced effects occur during a specific (embryonic day 6.5–9.5) gestational time frame. They also reported caffeine-induced changes in cardiac function, morphology, and gene expression consistent with hypertrophic cardiomyopathy in F2 animals whose gametes had been prenatally exposed to caffeine and the subsequent F3 generation that had never been directly exposed to caffeine (86), suggesting that caffeine induces heritable epigenetic changes in gene expression that can impact future generations.

Animals studies indicate that prenatal caffeine also alters vascular function in adult offspring. Serapiao-Moraes (84) reported increased blood pressure in adult mice that had been exposed to caffeine throughout the gestational period. This was accompanied by upregulation of renin expression, increased serum angiotensin II concentrations, and increased expression of ventricular angiotensin II receptors. In addition, Li et al. (87) reported potentiation of phenylephrine-induced increases in blood pressure and hyperresponsiveness to phenylephrine-induced contractile responses in mesenteric resistance arteries of adult rats that had been prenatally exposed to caffeine. These limited studies suggest that prenatal exposure to caffeine may induce stable changes in the expression of genes that regulate the renin-angiotensin system and constriction of vascular smooth muscle in resistance arteries, potentially increasing the risk of developing hypertension during adulthood.

In addition to its effects on contractile function, prenatal caffeine may also increase the risk of cardiovascular disease by promoting hypercholesterolemia. Guo et al. reported that adult rats that had been prenatally exposed to caffeine had increases in total cholesterol, low density lipoproteins (LDL), and apolipoprotein B expression following prenatal exposure to caffeine. These animals also had suppressed high density lipoprotein (HDL) and decreased expression of LDL receptors (88). These changes were accompanied by upregulation of hepatic genes that encode proteins involved in cholesterol synthesis (Hmg CoA reductase, Hmg CoA synthase 1, and sterol regulatory element binding factor 2) (21). These data suggest that prenatal caffeine may increase the propensity to develop cardiovascular disease during adulthood by enhancing cholesterol synthesis and altering cholesterol transport.

Widespread dietary consumption of caffeine makes it difficult to investigate the impact of prenatal caffeine on adult cardiovascular function in humans. We are unaware of any studies that have assessed the impact of maternal caffeine consumption on blood pressure, cardiac physiology, blood lipid profiles, or other cardiovascular parameters in human offspring. The widespread consumption of caffeine, prevalence of cardiovascular disease, and evidence of negative cardiovascular outcomes in animal models warrants further investigation to assess the potential long-term cardiovascular risks of prenatal caffeine exposure in humans.

## Potential Roles of Common Mechanisms

Despite having different molecular targets, CNS stimulants share some common effects on the fetus that may explain the mechanism by which they adversely impact the adult heart. Maternal use of cocaine (33), methamphetamine (27), and nicotine (73) suppress PKC-ε expression in the hearts of adult offspring, resulting in myocardial hypersensitivity to ischemic injury. All of these stimulants also decrease uterine blood flow (89–91), resulting in fetal hypoxia (92–94). Importantly, fetal hypoxia also induces methylation of the gene encoding PKC-ε and suppresses cardiac expression of this cardioprotective protein (95). Thus, the epigenetic attenuation of PKC-ε expression observed following gestational exposure to these stimulants might be secondary to decreased umbilical blood flow and fetal hypoxia rather than through a direct effect on the fetal heart or a CNS-mediated effect. In addition, maternal use of cocaine (96), methamphetamine (97), nicotine (98), and caffeine (99) increase circulating norepinephrine concentrations. α1-adrenergic receptor signaling increases methylation of the PKC-ε gene and suppresses its expression in the fetal heart (100). Thus, epigenetic reprogramming of PKC-ε expression and sensitization of the heart to ischemic injury may be the result of enhanced α1-adrenergic receptor signaling during the gestational period.

The ability of CNS stimulants to disrupt sleep is well-established. Disruption of sleep patterns and alterations in the circadian rhythm increase myocardial sensitivity to ischemia in rodents (101, 102) and are associated with an increased risk of myocardial infarction in humans (103). Elevated blood pressure, attenuation of the baroceptor reflex, and altered renal function have been reported in adult offspring of rats that were sleep deprived during pregnancy (104). Thus, the cardiovascular impact of prenatal exposure to CNS stimulants could be secondary to the ability of these agents to disrupt sleep patterns and circadian function during pregnancy.

Multiple studies have established a correlation between low birth weight and increased risk of cardiovascular diseases during adulthood (105–107). The mechanism by which this occurs is unclear. However, prenatal exposure to cocaine (108, 109), methamphetamine (110, 111), caffeine (112, 113), and nicotine (114, 115) are linked to low birth weight. Thus, the cardiovascular effects of these stimulants on the adult offspring may be secondary to low birth weight rather than a direct effect of the drugs on the developing cardiovascular system.

## Unanswered Questions and Future Directions

**Figure 1:**
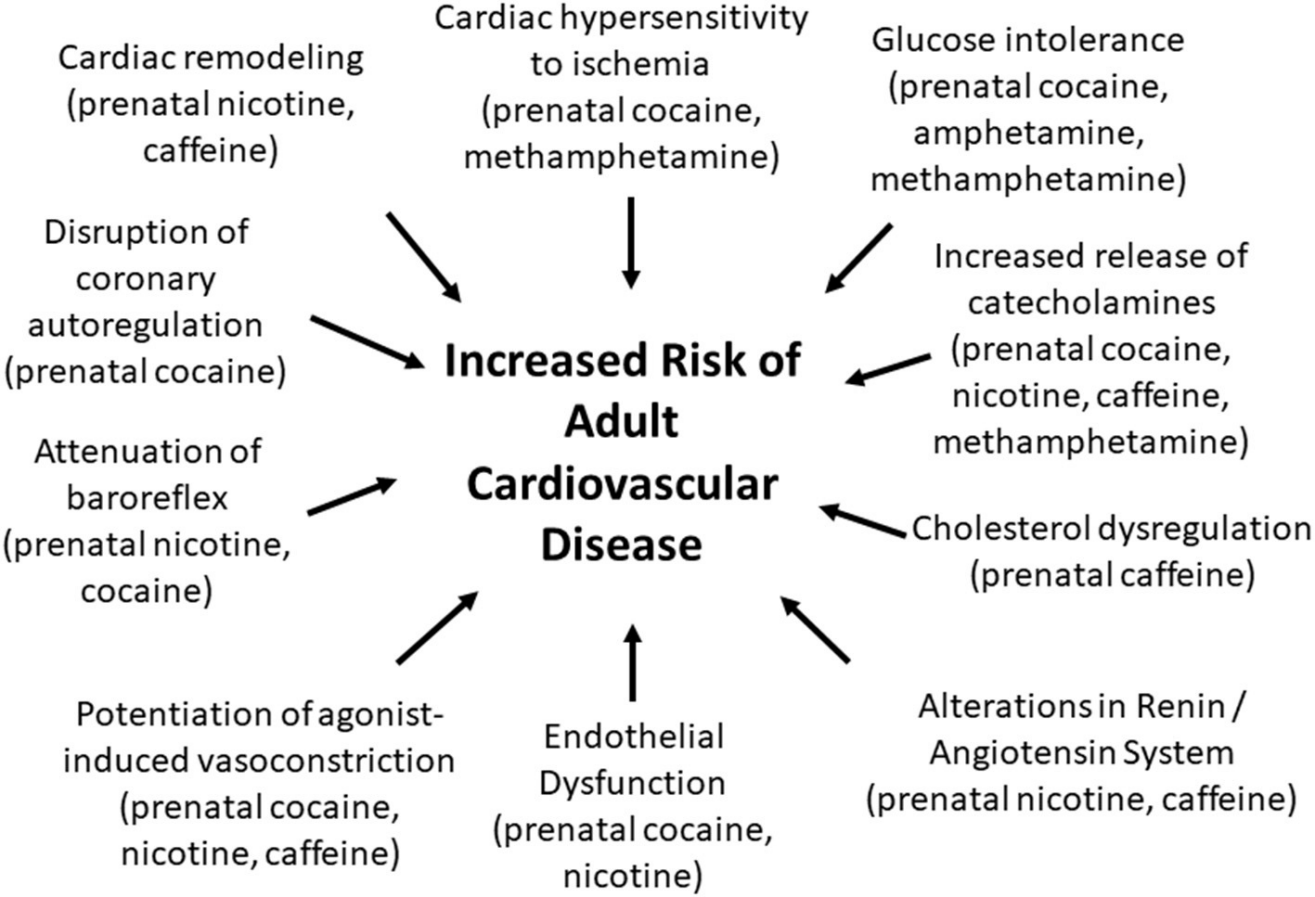
Prenatal exposure to CNS stimulants induces changes in the adult offspring that may increase the risk for developing cardiovascular diseases.

Fetal exposure to CNS stimulants can have a variety of negative cardiovascular consequences that extend into adulthood (Figure 1). However, there are significant gaps in our understanding of how prenatal stimulants impact the risk of developing cardiovascular disorders in adult humans. Most work in this area has been performed in rodent models with very few human studies. Thus, it is unclear whether the cardiovascular changes that have been reported in rodents that were prenatally exposed to CNS stimulants also occur in men and women. Another gap in our understanding is the fact that all animal studies reported up to this point have investigated the cardiovascular consequences of fetal exposure to CNS stimulants in relatively young adult animals (generally 1–5 months of age). Likewise, most human studies that have been performed have been limited to assessment of cardiovascular outcomes in children. In contrast, human cardiovascular disease is most prevalent in the older population. Longitudinal cardiovascular assessments in people who were prenatally exposed to CNS stimulants are needed to evaluate the extent to which prenatal exposure to CNS stimulants represents a cardiovascular risk factor in men and women. Studies that include geriatric individuals that were prenatally exposed to methamphetamine, cocaine, nicotine, and other stimulants are particularly needed since cardiovascular diseases are most prevalent in the geriatric population.

Polydrug use is common among both illicit drug users as well as those who consume tobacco products and dietary caffeine. Previous studies have demonstrated that smokers drink more coffee than non-smokers and that tobacco and coffee consumption tend to occur simultaneously (116–118). A study in the United Kingdom found a similar relationship between nicotine use and tea consumption (119). In contrast, animal models up to this point have investigated the effects of single stimulants in isolation. We do not know whether concurrent fetal exposure to multiple stimulants (i.e., nicotine and caffeine; methamphetamine and cocaine; etc.) or concurrent use of stimulants with other drug classes (i.e., opioids) produces cardiovascular effects in the offspring that qualitatively or quantitatively differ from fetal exposure to stimulants used in isolation. Furthermore, studies investigating the impact of fetal exposure to CNS stimulants on adult cardiovascular function have been limited to the agents discussed in this review. Notably, prescriptions for 18.6 million tons of methylphenidate were filled in the United States in 2016 (120). However, we are unaware of any studies that have investigated the potential impact of fetal methylphenidate exposure on adult cardiovascular function. There is also a lack of data regarding the impact of 3,4-methylendioxymethamphetamine (“ecstasy”), synthetic cathinones (“bath salts”), or other “club drugs.” We speculate that fetal exposure to these stimulants may have effects on the adult cardiovascular system that are similar to those described for cocaine and methamphetamine.

Animal studies provide evidence that fetal exposure to CNS stimulants leads to changes in the expression of protein kinase C-ε (27, 32, 73), myosin heavy chain (86), large-conductance calcium-activated potassium (BKca) channels (121), DNA methyltransferase (122), and other genes in the adult heart through mechanisms that involve DNA methylation (122), microRNA (121, 123), and potentially other epigenetic mechanisms. Importantly, some of these changes are sex-dependent (27, 28, 72), indicating that these stimulants impact the developing male and female cardiovascular systems differently. However, our knowledge of these changes is limited to a small number of selected genes. We are unaware of any transcriptome-wide analyses of myocardial or vascular changes in gene expression or epigenetic modifications in adult animals or humans following prenatal exposure to CNS stimulants. Such studies are likely to provide a more comprehensive understanding of the impact of prenatal stimulants on the adult cardiovascular system.

It is unclear whether the cardiovascular effects that have been observed in adult offspring following prenatal exposure to CNS stimulants result from a direct effect of these drugs on the developing heart and vasculature or if they are secondary to stimulant-induced changes in the CNS. Previous work has demonstrated that prenatal exposure to cocaine (5), nicotine (124, 125), and methamphetamine (2, 3) lead to sex-dependent changes in the CNS of adult rodents. The ability of these drugs to induce sex-dependent changes in the developing nervous system that subsequently impact cardiovascular function could potentially provide a mechanism by which CNS stimulants induce sex dependent changes in the adult heart and vasculature. Further work is needed to understand the mechanism by which prenatal stimulants induce sex-dependent changes in the adult cardiovascular system and whether these changes result from direct effects on the heart or are secondary to alterations in CNS function.

## Conclusions

In conclusion, there is growing evidence that fetal exposure to cocaine, methamphetamine, nicotine, and caffeine negatively impact the heart and vasculature through a variety of mechanisms that may lead to an increased risk of developing cardiovascular disease during adulthood (Figure 1; Supplemental Table 1). Our understanding of these effects is almost exclusively limited to animal models. Long term human studies are needed to determine whether men and women who were prenatally exposed to cocaine, methamphetamine, nicotine, and other CNS stimulants are at increased risk of developing myocardial infarction, hypertension, diabetes, and other cardiometabolic disorders.

## Author Contributions

This manuscript was written by BR.

## Conflict of Interest

The author declares that the research was conducted in the absence of any commercial or financial relationships that could be construed as a potential conflict of interest.
