# Supplementary material for: Does Prenatal Exposure to CNS Stimulants Increase the Risk of Cardiovascular Disease in Adult Offspring?
Source: Front Cardiovasc Med. 2021 Mar 4;8:652634. doi: 10.3389/fcvm.2021.652634 (PMC7969998; doi:10.3389/fcvm.2021.652634)
Supplement: Supplementary file 1 [file Table_1.DOCX]

**Supplemental Table 1. Summary of animal studies investigating the cardiovascular impact of prenatal exposure to CNS stimulants.**

| **Model of Prenatal Stimulant Exposure** | **Cardiovascular Impact on Adult Offspring** | **Reference** |
| --- | --- | --- |
| **Cocaine** |  |  |
| Pregnant rats received cocaine injections (15 mg/kg, i.p.) twice daily during gestational days 15-21 | Cardiac hypersensitivity to ischemic injury (male offspring only) | 28 |
| Pregnant mice received cocaine injections (20 mg/kg/ day, i.p.) on gestational days 14.5-16.5 | Lifelong impairment of insulin production and glucose intolerance (female offspring only) | 13 |
| Pregnant rats received cocaine injections (30 mg/kg/day, i.p.) during gestational days 15-21 | Decreased pressure-dependent myogenic contractions in isolated coronary arteries | 26 |
| Pregnant rats received cocaine injections (30 mg/kg/day, i.p.) during gestational days 15-21 | Loss of cardioprotection induced by ischemic preconditioning (male offspring only) | 31 |
| Pregnant rats received cocaine injections (30 mg/kg/day, i.p.) during gestational days 15-21 | Decreased myocardial expression of PKC-ε resulting from methylation of the promoter region of the PKC-ε gene (male offspring only) | 32,33, 45 |
| Pregnant rats received cocaine injections (30 mg/kg/day, i.p.) during gestational days 15-21 | Potentiation of norepinephrine-induced increase in blood pressure (male offspring only)  Attenuation of endothelium-dependent relaxation and potentiation of norepinephrine-induced contraction of isolated mesenteric artery rings (male offspring only)  Decreased sensitivity of the baroceptor reflex (male offspring only) | 34 |
| **Methamphetamine** |  |  |
| Pregnant rats received methamphetamine injections (5 mg/kg/day, s.c.) on gestational days 1-21 | Cardiac hypersensitivity to ischemic injury (female offspring only)  Decreased myocardial expression of PKC-ε (female offspring only) | 27 |
| Pregnant mice received methamphetamine injections (10 mg/kg, i.p.) on gestational days 14.5-16.5 | Impairment of insulin production and glucose intolerance (female offspring only) | 13 |
| **Nicotine** |  |  |
| Pregnant rats received nicotine injections (1mg/kg/day, i.p.). throughout pregnancy. Injections of the dams continued until the pups were weaned on postnatal day 21 | Increased basal blood pressure | 56 |
| Pregnant mice consumed nicotine in their drinking water (200 μg/ml) throughout gestation. Nicotine treatment was continued until the pups were weaned at day 21 | Increased basal blood pressure | 58 |
| Nicotine was delivered to pregnant rats through an osmotic pump (6 mg/kg/day) starting on gestational day 4 and ending on postnatal day 10 | Potentiation angiotensin II-induced increases in blood pressure and potentiation of vasoconstriction in isolated aorta and mesenteric arteries (male offspring only)  Increased expression of angiotensin II receptors in vascular smooth muscle and thickening of tunica media in arterial wall | 59, 60 |
| Nicotine was delivered to pregnant rats through an osmotic pump (6 mg/kg/day) starting on gestational day 4 and ending on postnatal day 10 | Increased oxidative stress in arterial wall  Attenuation of endothelium-dependent vasodilation  Potentiation of angiotensin II-induced vasoconstriction | 24 |
| Nicotine was delivered to pregnant rats through an osmotic pump (6 mg/kg/day) starting on gestational day 4 and ending on postnatal day 10 | Cardiac hypersensitivity to ischemic injury  Decreased myocardial expression of PKC-ε | 72 |
| Pregnant rats received nicotine injections (1.5 mg/kg, s.c.) on gestational days 3-21 | Cardiac fibrosis, decreased ejection fraction, and decreased fractional shortening  Increased circulating concentrations of norepinephrine and epinephrine | 63 |
| **Caffeine** |  |  |
| Pregnant mice received caffeine injections (20 mg /kg/day, s.c.) throughout gestation | Cardiac hypertrophy, increased angiotensin II expression, increased angiotensin II receptor expression, increased angiotensin converting enzyme expression, increased renin expression, increased blood pressure | 84 |
| Pregnant mice received 20 mg/kg caffeine (i.p.) on gestational day 8.5 | Increased ventricular wall thickness, decreased cardiac output, methylation of genes involved in cardiac hypertrophy | 85 |
| Pregnant mice received 20 mg/kg/ day i.p) once daily from gestational day 6.5-9.5 | Dilated cardiomyopathy at 1 year of age.  Impaired β-adrenergic receptor signaling | 85, 86 |
| Pregnant rats received caffeine injections (20 mg/kg caffeine twice / day) throughout gestation | Potentiation of phenylephrine-induced increase in blood pressure and potentiation of phenylephrine-induced contraction of mesenteric arteries | 87 |
| Pregnant rats received 120 mg caffeine /kg / day orally from gestational day 11 until the pups were delivered | Increased total cholesterol increased low density lipoproteins, and increased apolipoprotein B expression  Decreased expression of high density lipoproteins, decreased expression of low density lipoprotein receptors  Increased expression of hepatic enzymes that regulate cholesterol synthesis | 88 |
